# Skin health and climate change in Africa: an overlooked intersection and call for action

**DOI:** 10.1093/inthealth/ihaf147

**Published:** 2025-12-18

**Authors:** Olufolakemi M Cole-Adeife, Ayesha O Akinkugbe, Olusola O Ayanlowo, Adewunmi Akingbola, Abel N Onunu

**Affiliations:** Dermatology Unit, Dept. of Medicine, Lagos State University Teaching Hospital/Lagos State University College of Medicine, Lagos 100271 Nigeria; Dermatology Unit, Department of Medicine, Lagos University Teaching Hospital/College of Medicine, University of Lagos, PMB 12003, Lagos, Nigeria; Dermatology Unit, Department of Medicine, Lagos University Teaching Hospital/College of Medicine, University of Lagos, PMB 12003, Lagos, Nigeria; Department of Public Health, University of Cambridge, Cambridgeshire CB2 1ST, UK; Vice Chancellor’s Office, Federal University of Medical and Health Sciences, Kwale 322101 Delta State, Nigeria

**Keywords:** Africa, climate change, dermatology, environmental health, skin diseases, skin health

## Abstract

Climate and environmental change are increasingly affecting skin health across Africa, but this remains under-recognised in global health discussions. Rising temperatures, ultraviolet radiation, pollution, flooding, deforestation, and droughts are reshaping the epidemiology of many cutaneous infections, inflammatory conditions, and malignancies. Unregulated cosmetic and antiseptic skincare products further compound both cutaneous and environmental harm. Addressing these emerging challenges is essential for health equity and resilience across the continent's rapidly evolving landscape. The recent World Health Assembly resolution on skin diseases as a global public health priority affirms this need to include dermatology and skin health in climate and environmental health policies.

Climate change and environmental degradation are increasingly recognised as major public health threats, yet their effects on skin health remain underacknowledged and understudied, particularly in Africa.^1^ The skin, our first line of defence and a visible marker of internal and environmental well-being, is uniquely vulnerable to shifts in temperature, humidity, air quality and ultraviolet (UV) radiation.^2,3^ Across Africa’s diverse climates, from the arid Sahel to the humid coastal belts, rapid ecological transitions are already influencing the burden and pattern of dermatological diseases.^2,3^ From urban pollution to flooding and deforestation, the skin, our outermost cover, also bears some of the brunt of ecological disruption, warranting urgent scientific and policy attention.^2,3^

Rising temperatures and intensifying UV radiation have increased rates of photodamage, photoaging and non-melanoma skin cancers.^3^ These risks are greatest among people with albinism, outdoor labourers, nomadic populations and individuals who engage in skin bleaching. Warmer, more humid environments also favour dermatomycoses and are associated with increased yeast (e.g. tinea versicolor and cutaneous candidiasis) and dermatophyte (e.g. trichophyton mentagrophytes) infections, including the emergence of recalcitrant strains, across the continent.^2,3^ Warmer temperatures favour the vectors of several tropical diseases, such as leishmaniasis and dengue, many of which have cutaneous manifestations.^2,3^

In major cities such as Lagos, Nairobi, Johannesburg and Accra, increasing air pollution from traffic, industrial emissions, hydrocarbon generators and bush burning contributes to acneiform eruptions, atopic and seborrhoeic dermatitis, pigmentary disorders and premature skin ageing.^3,4^ Anecdotal reports of rising incidence of cutaneous T-cell lymphoma in Lagos, hypothesised to be connected to environmental toxins, stress the need for dermato-epidemiological studies.

Climatic extremes often amplify the burden of skin-related neglected tropical diseases. Recurrent droughts and water scarcity in some areas increase the prevalence of scabies and dracunculiasis.^2,3^ Drought-related malnutrition may increase noma and leprosy incidences, as well as other nutritional dermatoses such as pellagra, angular cheilitis and phrynoderma.^2,3^ Heavy rains and flooding favour bacterial skin infections, Buruli ulcer, deep fungal mycoses, tungiasis, mycetoma and vector-driven diseases such as leishmaniasis, onchocerciasis and lymphatic filariasis.^2,3^

Deforestation, mining and urban encroachment on wildlife habitats across Central and West Africa, driven by population surges, have intensified human contact with animal reservoirs of zoonotic pathogens.^5^ The resurgence of mpox exemplifies this interface. Although waning smallpox herd immunity remains a key factor, expanding agriculture, bushmeat hunting and settlement within degraded forests increase contact with zoonotic diseases.^5^

Furthermore, unregulated antiseptic soaps and skin-lightening products containing potent topical corticosteroids or heavy metals such as mercury and arsenic contribute to both cutaneous injury and environmental contamination.^6^ Such agents disrupt the skin microbiome, promote antimicrobial resistance, pollute the soil and water and create a cycle of harm to humans, animals and ecosystems.

Across Africa, subtle yet significant shifts in dermatologic disease patterns are evident. In some regions, prolonged drought and rising UV intensity are associated with a higher incidence of actinic keratoses and skin cancers in persons with albinism.^2,3,7^ In polluted megacities, inflammatory dermatoses (acne, seborrhoeic dermatitis, atopic dermatitis and psoriasis) and pigmentary disorders are increasingly prevalent.^3,7^ In the Niger Delta and mining zones of Zambia and Angola, chemical and hydrocarbon exposures contribute to irritant and pigmentary dermatoses. Yet these changes remain poorly quantified because skin diseases are rarely included in environmental or climate-health surveillance systems.^1,3^ Their omission perpetuates underinvestment in prevention, research and health workforce training.^1,2^

Safeguarding skin health to improve quality of life, reduce healthcare expenditure and improve productivity must become a pillar of Africa’s climate-resilient health agenda.^2,3,7^ Ministries of health, environment and science should collaborate to integrate dermatologic indicators into national climate-and-health observatories,^3,7^ promote sun-safety education and equitable access to affordable photoprotective measures, strengthen the regulation of the cosmetic and skincare industries to eliminate environmentally hazardous formulations and packaging and expand research into environmental dermatology, exposome-related skin disease and the African skin microbiome.

Dermatologists, clinicians and public health practitioners are uniquely positioned to detect climate-related shifts in disease patterns. They can guide adaptation strategies through multidisciplinary collaborative research to document the complex links between climate, pollution and skin diseases.^2,3^

In May 2025, the World Health Assembly adopted the first general resolution on skin diseases, recognising that >1 billion people worldwide are affected by skin conditions that cause disability, stigma and reduced quality of life.^7,8^ The resolution urges member states to strengthen skin health within universal health coverage, enhance access to dermatologic care and address the environmental and social determinants of skin disease.^7,8^ This global mandate offers a timely opportunity for African governments and regional bodies to integrate skin health considerations into environmental and climate resilience policies.^2,3,7,8^

The skin often reflects both personal and climate health. As Africa, along with the rest of the world, faces intensifying heat, pollution, deforestation and ecological instability, safeguarding skin health is a fundamental component of health equity that is often overlooked.^2,3,7^ Many African nations are struggling to maintain health and social services due to weak economies, and thus remain ill-prepared and most vulnerable to the dermatologic and broader health consequences of climate change.^1–3^ Addressing skin health in the context of climate change in Africa is in the interest of the continent’s 1.5 billion people.

## Data Availability

No new data were generated or analysed for this commentary.
